# Supplementary material for: Diversity of visual inputs to Kenyon cells of the Drosophila mushroom body
Source: Nat Commun. 2024 Jul 7;15:5698. doi: 10.1038/s41467-024-49616-z (PMC11228034; doi:10.1038/s41467-024-49616-z)
Supplement: Supplementary file 1 — Supplementary Information [file 41467_2024_49616_MOESM1_ESM.pdf]

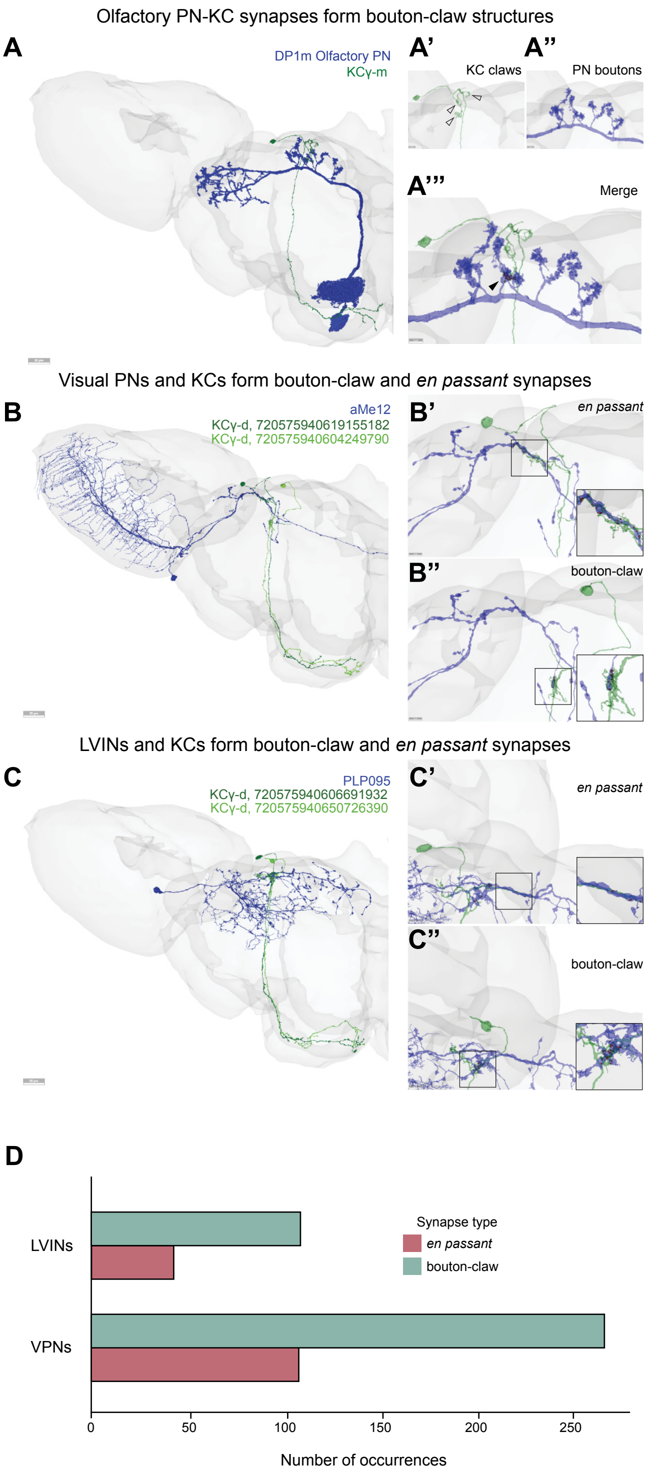

Supplementary Figure 1. KCγ-d's form bouton-claw synapses and *en passant* synapses with VPNs and LVINs.
A. Olfactory projection neurons and olfactory Kenyon cells form bouton-claw synapses typical of the olfactory calyx.
(A') KCγ-m cell with dendritic claws indicated with open arrowheads. (A") Zoom in showing PN boutons.
(A"') Merge of A' and A" with bouton claw contact indicated by arrowhead. Red dots, synapses shared between the PN and KC.
Scale bar for A, 20μm. Scale bar for A'-A"', 5μm.
B. Example VPN, aMe12, forms *en passant* synapses with one KCγ-d (B') and a bouton-claw synapse with another KCγ-d (B").
Insets show zoom in of the boxed regions. Synapse locations displayed as red dots. Scale bar for B, 20μm. Scale bar for B'-B", 5μm.
C. Example LVIN, PLP095, forms *en passant* synapses with one KCγ-d (C') and a bouton-claw synapse with another KCγ-d (C").
Insets show a zoom in of the boxed regions. Synapse locations displayed as red dots. Scale bar for C, 20μm. Scale bar for C'-C", 5μm.
D. Number of connections between presynaptic visual inputs and postsynaptic KCγ-d's that were putatively classified as bouton-claw or *en passant* synapses types using our synapse type detection algorithm (see Methods). Distribution of putative synapse type is shown separately for LVIN inputs and direct VPN inputs to KCγ-d's.
Source data are provided as a Source Data file.
Supplementary Figure 1A-C neuron skeletons and neuropil volumes adapted with permission under CC BY-NC 4.0 license (https://creativecommons.org/licenses/by-nc/4.0/) from Dorkenwald et al. Neuronal wiring diagram of an adult brain. bioRxiv 2023.06.27.546656 (2023) doi:10.1101/2023.06.27.546656 and Schlegel et al. Whole-brain annotation and multi-connectome cell typing quantifies circuit stereotypy in Drosophila. bioRxiv 2023.06.27.546055 (2023) doi: https://doi.org/10.1101/2023.06.27.546055.

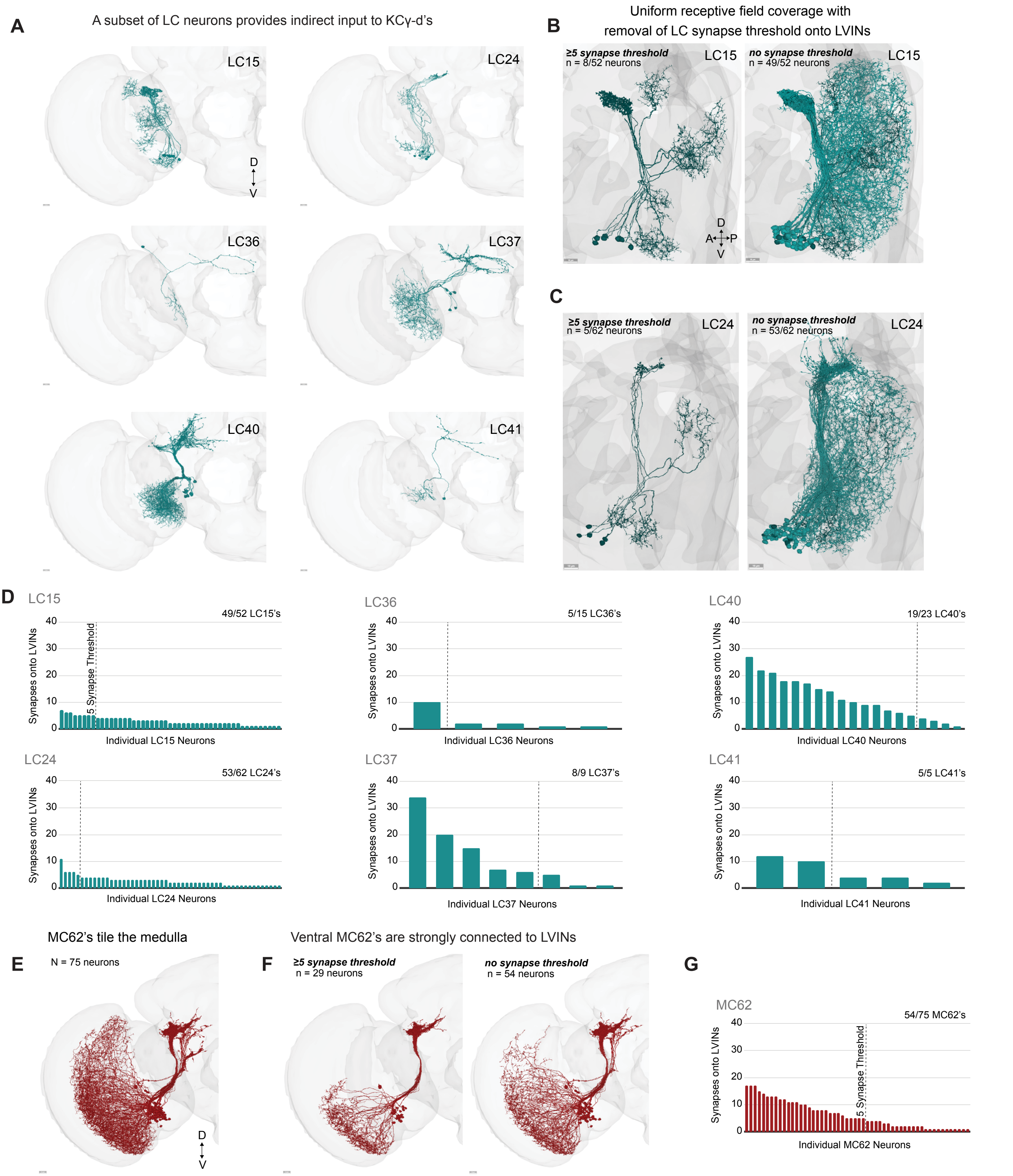

Supplementary Figure 2. Releasing the standard synapse threshold unmasks patterns of indirect visual input.
A. A subset of lobula columnar neurons (LCs) provide indirect inputs to KCγ-d's via LVINs. Scale bars, 10μm.
B. Left, 8/52 LC15 neurons form ≥5 synapses with LVINs, creating a sporadically filled receptive field.
Right, when the synapse threshold is removed, 49/52 LC15's are now found to synapse with LVINs, creating a uniform coverage of the receptive field. Scale bars, 10μm.
C. Left, 5/62 LC24 neurons form ≥5 synapses with LVINs. Right, when the synapse threshold is removed, 53/62 LC24's are now found to synapse with LVINs. Scale bars, 10μm.
D. Number of synapses made by individual unique LC neurons onto LVINs. Dashed lines indicate the 5-synapse threshold. The fraction of total LCs that make at least one synapse with an LVIN is indicated in the upper right of each graph.
E. The population of 75 MC62 neurons annotated in FlyWire have dendrites that tile the entire medulla. Scale bar, 10μm.
F. Left, 29/75 MC62 neurons synapse with LVINs when using a ≥5 synapse threshold. These neurons cover the ventral half of the medulla.
Right, when the synapse threshold is removed 54/75 MC62's are now found to synapse with LVINs but coverage is lacking in the dorsal-most region of the medulla.
Scale bars, 10μm.
G. Number of synapses made by individual unique MC62 neurons onto LVINs. Dashed line indicates the 5-synapse threshold.
The fraction of total MC62s that make at least one synapse with an LVIN is indicated in the upper right of the graph.
Source data are provided as a Source Data file.
Supplementary Figure 2A-C, E-F neuron skeletons and neuropil volumes adapted with permission under CC BY-NC 4.0 license (https://creativecommons.org/licenses/by-nc/4.0/) from Dorkenwald et al. Neuronal wiring diagram of an adult brain. bioRxiv 2023.06.27.546656 (2023) doi:10.1101/2023.06.27.546656 and Schlegel et al. Whole-brain annotation and multi-connectome cell typing quantifies circuit stereotypy in Drosophila. bioRxiv 2023.06.27.546055 (2023) doi: https://doi.org/10.1101/2023.06.27.546055.

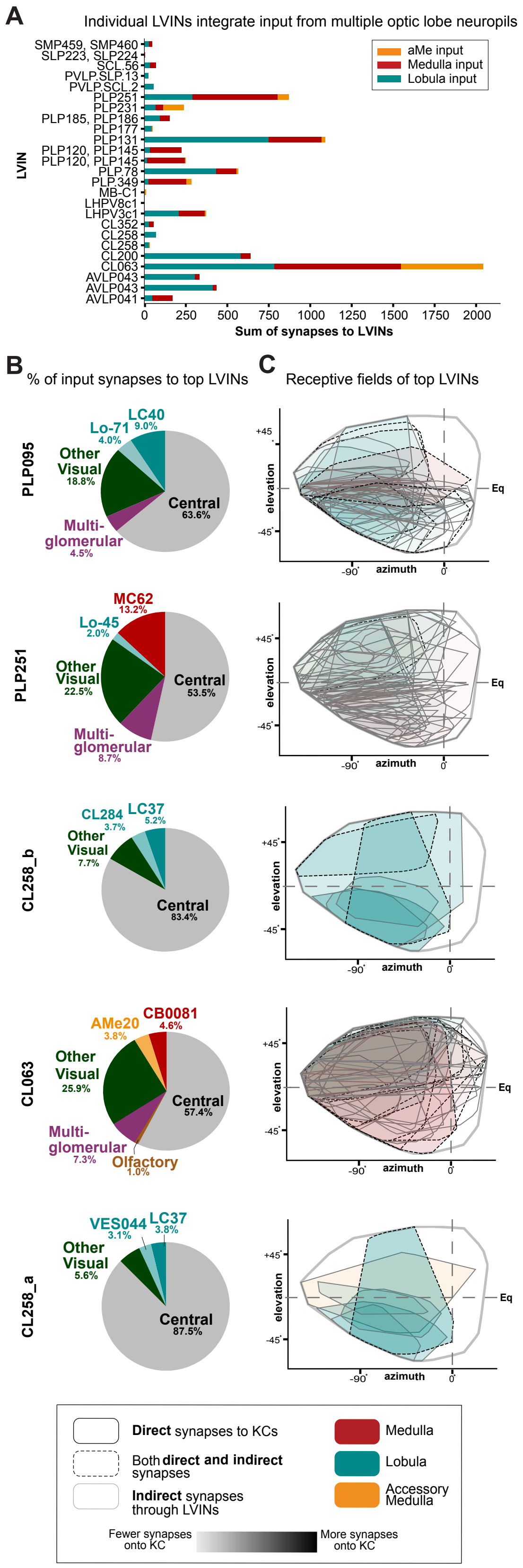

Supplementary Figure 3. LVINs integrate diverse sets of inputs.
A. Proportion of synapses from the primary sources of optic lobe input (aMe, medulla, and lobula) onto each individual LVIN. Most LVINs integrate a combination of input from different optic lobe neuropils. Note that LHPV8c1 only receives 5 synapses from a single lobula neuron, which due to the scale of the y-axis is not visible.
B. Percentages of direct synaptic input received from the top two input VPN classes as well as all other visual inputs to the top 5 LVINs shown in (B).
Also shown are the percentages of direct synaptic input from other sensory origins (multi-glomerular, olfactory) and the central brain.
C. The effective receptive fields of the top 5 LVINs (selected by considering the sum of synapses onto KCγ-d's) are a combination of individual input VPN receptive fields.
Color indicates source input neuropil and shading indicates the strength of connectivity (sum of synapses) onto the LVIN.
LVINs receive multiple visual inputs with distributed weights.
Source data are provided as a Source Data file.

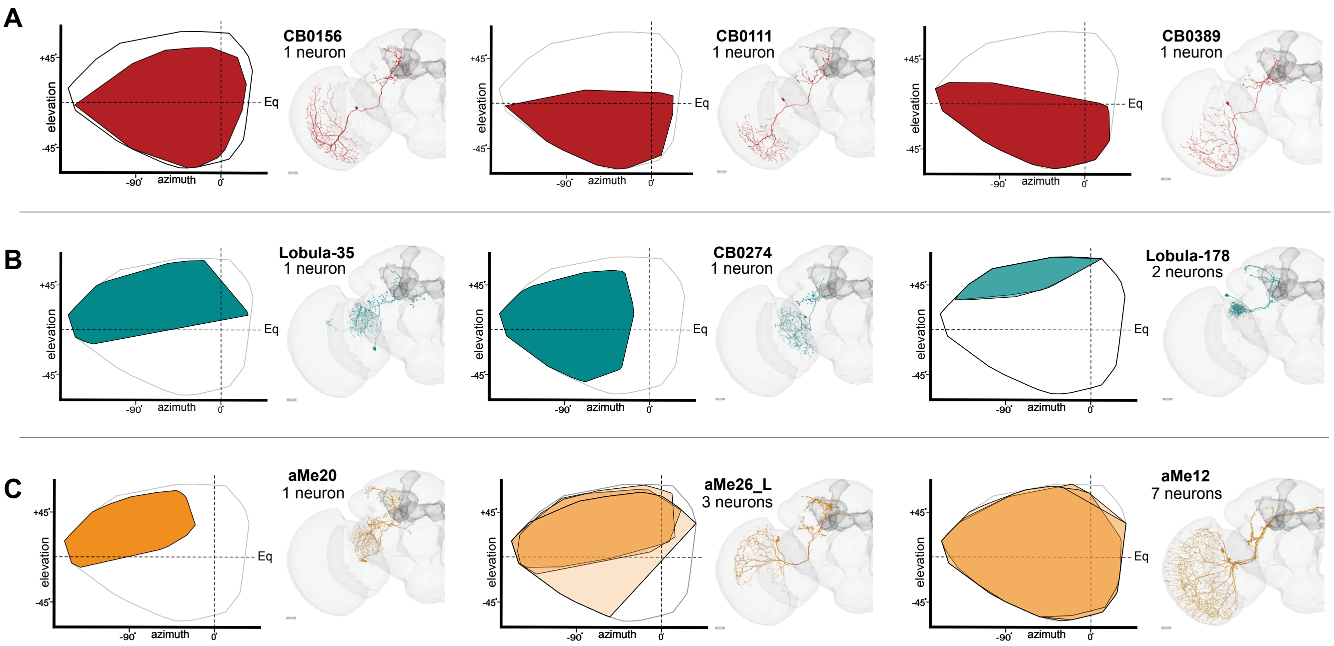

Supplementary Figure 4. Receptive fields of top morphological classes that contact KCγ-d's.
A. Estimated receptive fields (Mollweide projections of ommatidial viewing angles) of the top three VPN input classes that carry visual information from the medulla to KCγ-d's. For each morphological class, the receptive fields of individual neurons are overlaid, and shading indicates what proportion of the class's total synaptic input to the KCγ-d population is accounted for by that neuron.
B. Estimated receptive fields as in (A) of the top three VPN input classes that carry visual information from the lobula to KCγ-d's.
C. Estimated receptive fields as in (A) of the top three VPN input classes that carry visual information from the accessory medulla to KCγ-d's.
Source data are provided as a Source Data file.
Supplementary Figure 4 neuron skeletons and neuropil volumes adapted with permission under CC BY-NC 4.0 license (https://creativecommons.org/licenses/by-nc/4.0/) from Dorkenwald et al. Neuronal wiring diagram of an adult brain. bioRxiv 2023.06.27.546656 (2023) doi:10.1101/2023.06.27.546656 and Schlegel et al. Whole-brain annotation and multi-connectome cell typing quantifies circuit stereotypy in Drosophila. bioRxiv 2023.06.27.546055 (2023) doi: https://doi.org/10.1101/2023.06.27.546055.

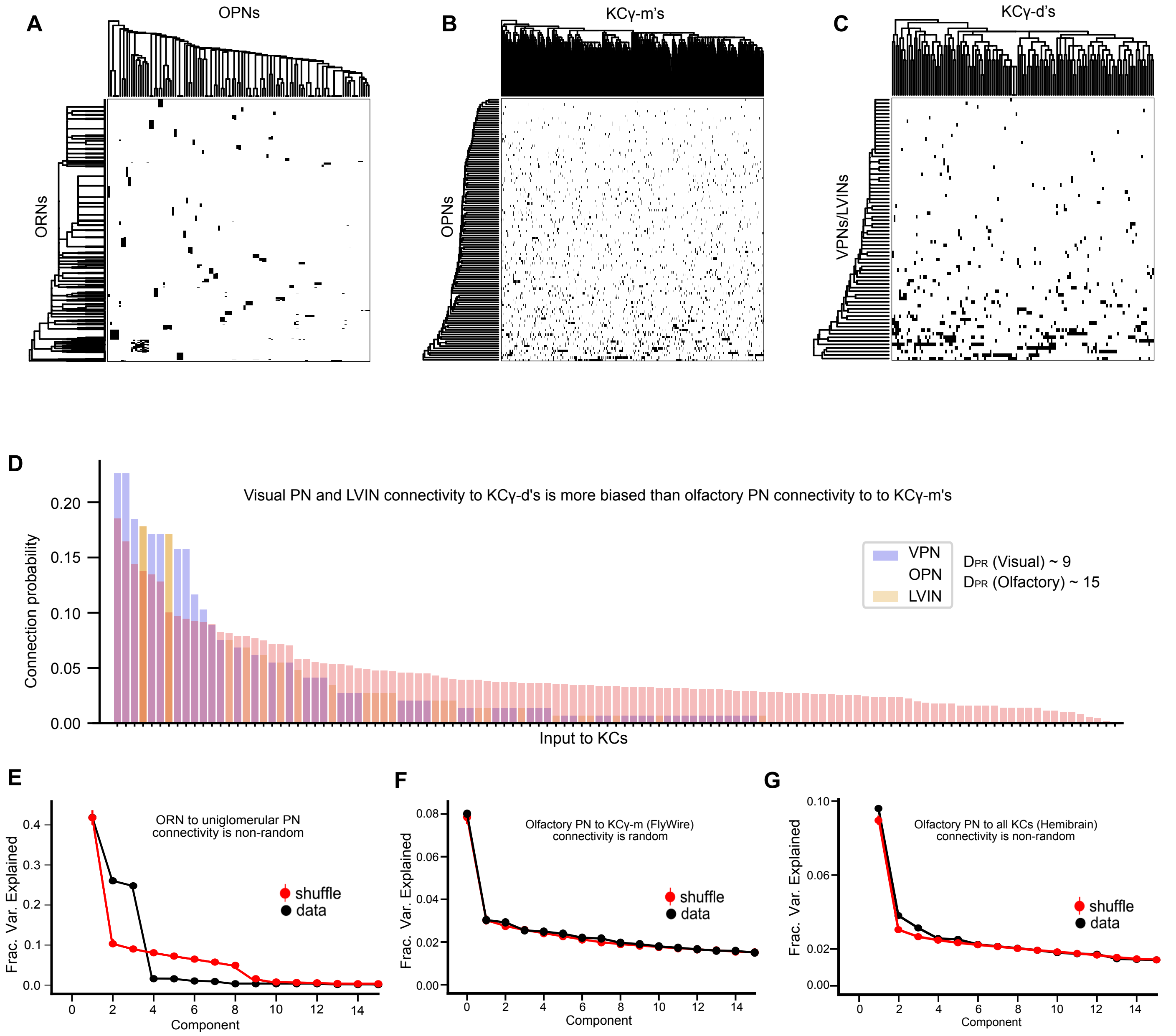

Supplementary Figure 5. Olfactory and visual PNs display analogous patterns of connectivity with Kenyon cells.
A. Olfactory receptor neurons (ORNs, rows) make largely discrete connections with uniglomerular olfactory projection neurons (OPNs, columns). Connections were binarized using a 5 synapse threshold. Black cells indicate connections with ≥5 synapses. Rows and columns are hierarchically clustered based on partner similarity.
B. Heatmap showing binarized Uniglomerular OPN-KCγ-m synaptic connections.
C. Heatmap showing binarized VPN/LVIN-KCγ-d synaptic connections.
D. Sorted connection probabilities of individual VPNs (blue) and individual LVINs (orange) onto the KCγ-d population compared to the sorted connection probabilities of individual OPNs (red) onto the KCγ-m population. Top VPNs and LVINs have larger, biased connection probabilities onto the KCγ-d population when compared to the top OPNs connecting to the KCγ-m population. This is supported by a lower dimensionality for visual connectivity compared to olfactory connectivity (estimated by computing the participation ratio, DPR) despite a smaller number of visual inputs.
E. Principal components analysis of olfactory receptor neuron (ORN) to olfactory projection neuron (OPN) connectivity. Red circles and bars represent mean and 95% confidence intervals for variance explained by the principal components of shuffled connectivity matrices. Top PC components account for higher proportions of variance when compared to shuffled matrices preserving ORN connection probability and number of inputs to each OPN, indicating structure in connectivity.
F. Principal components analysis of uniglomerular olfactory projection neuron to KCγ-m connectivity (FlyWire dataset). Top PC components do not account for higher proportions of variance when compared to shuffled matrices preserving OPN connection probability and number of inputs to each KCγ-m.
G. Principal components analysis of connectivity from uniglomerular olfactory projection neurons to KCs in all MB lobes from the hemibrain connectome. Top PC components account for higher proportions of variance when compared to shuffled matrices preserving OPN connection probability and number of inputs to each KC, indicating some structure in this connectivity.
Data in this figure examine connectivity in the left hemisphere, using a ≥5 synapse threshold.
Source data are provided as a Source Data file.

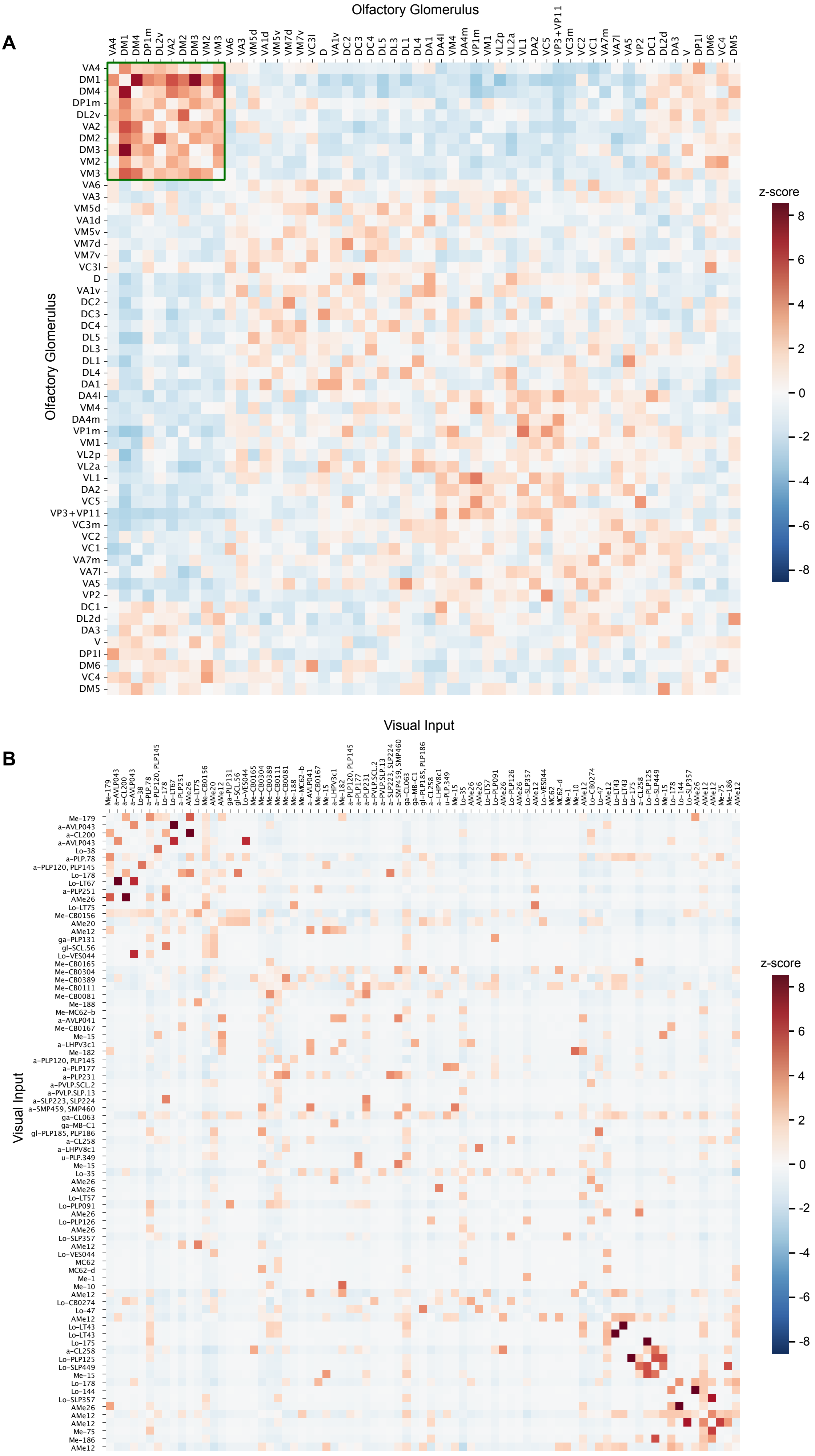

Supplementary Figure 6. Conditional input analysis does not reveal significant structure in direct VPN and LVIN connectivity to KCγ-d's.
A. Result of a conditional input analysis (Zheng et al. 2020) applied to the olfactory uniglomerular PN (condensed by input glomerulus) to KC connectivity observed in the FlyWire dataset. The value in each cell of the matrix is a z-score for the observed conditional connectivity (sum of inputs to the KC cell population from the column glomerulus given input from the row glomerulus) from a null distribution of 1,000 random shuffles. A positive value (red) and negative value (blue) indicate larger and smaller conditional input counts respectively compared to random models. The group of glomeruli outlined by a green border was identified in Zheng et al. as structured input related to food-sensing glomeruli.
B. As in (A), but for the observed direct VPN and LVIN to KCγ-d connectivity in the FlyWire dataset.
Source data are provided as a Source Data file.

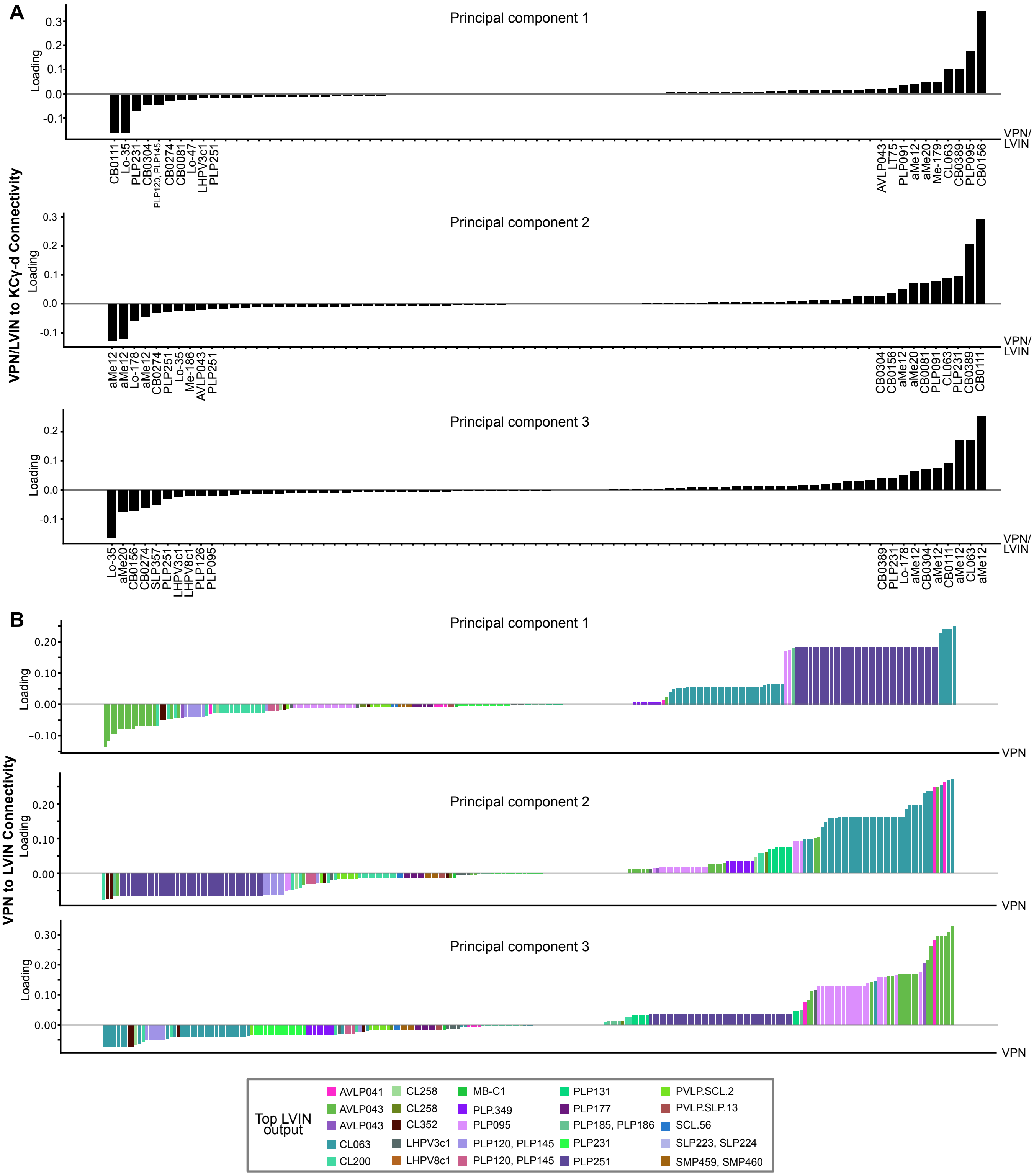

Supplementary Figure 7. Top PC loadings reflect input connection probabilities in VPN/LVIN to KCγ-d connectivity and top LVIN output identity in VPN to LVIN connectivity.

A. Principal component loadings, or the correlation between each input variable (VPN or LVIN) and a principal component, for the VPN/LVIN to KCγ-d connectivity shown in Figure 5D. The top three PC loadings are shown. VPNs or LVINs with high correlation or inverse correlation with each PC are labeled.

B. Principal component loadings, or the correlation between each input variable (each VPN) and a principal component, for the VPN to LVIN connectivity shown in Figure 3F. The top three PC loadings are shown. Each VPN is colored based on the LVIN it makes the most synapses with.

Data in this figure examine connectivity in the left hemisphere, using a ≥5 synapse threshold.

Source data are provided as a Source Data file.
