# Supplementary material for: Diversity of visual inputs to Kenyon cells of the Drosophila mushroom body
Source: Nat Commun. 2024 Jul 7;15:5698. doi: 10.1038/s41467-024-49616-z (PMC11228034; doi:10.1038/s41467-024-49616-z)
Supplement: Supplementary file 3 — Reporting Summary [file 41467_2024_49616_MOESM3_ESM.pdf]

nature portfolio

Corresponding author(s): Dr. Rudy Behnia (rb3161@columbia.edu), Dr. Josie Clowney (jclowney@umich.edu)

Last updated by author(s): May 14, 2024

Reporting Summary

Nature Portfolio wishes to improve the reproducibility of the work that we publish. This form provides structure for consistency and transparency in reporting. For further information on Nature Portfolio policies, see our Editorial Policies and the Editorial Policy Checklist.

Statistics

For all statistical analyses, confirm that the following items are present in the figure legend, table legend, main text, or Methods section.

n/a	Confirmed	
☐	☒	The exact sample size (*n*) for each experimental group/condition, given as a discrete number and unit of measurement
☐	☒	A statement on whether measurements were taken from distinct samples or whether the same sample was measured repeatedly
☐	☒	The statistical test(s) used AND whether they are one- or two-sided<br>*Only common tests should be described solely by name; describe more complex techniques in the Methods section.*
☐	☒	A description of all covariates tested
☐	☒	A description of any assumptions or corrections, such as tests of normality and adjustment for multiple comparisons
☐	☒	A full description of the statistical parameters including central tendency (e.g. means) or other basic estimates (e.g. regression coefficient) AND variation (e.g. standard deviation) or associated estimates of uncertainty (e.g. confidence intervals)
☐	☒	For null hypothesis testing, the test statistic (e.g. *F*, *t*, *r*) with confidence intervals, effect sizes, degrees of freedom and *P* value noted<br>*Give P values as exact values whenever suitable.*
☒	☐	For Bayesian analysis, information on the choice of priors and Markov chain Monte Carlo settings
☒	☐	For hierarchical and complex designs, identification of the appropriate level for tests and full reporting of outcomes
☐	☒	Estimates of effect sizes (e.g. Cohen's *d*, Pearson's *r*), indicating how they were calculated

Our web collection on statistics for biologists contains articles on many of the points above.

Software and code

Policy information about availability of computer code

Data collection	Neurons in the FlyWire connectome (v630) were queried using a combination of open source software and online packages - the natverse R package (R version 4.3.1 (2023-06-16), RStudio Version 2023.06.1+524) and the accompanying fafbseg library; the NAVis python library version 1.3.1 (https://navis.readthedocs.io/en/latest/index.html) and accompanying fafbseg-py package version 1.13.0 (https://github.com/navis-org/fafbseg-py); the flybrains package version 0.2.6 (https://pypi.org/project/flybrains/) and the online Connectome Data Explorer (Codex, https://codex.flywire.ai/). Data was queried from the hemibrain dataset using the neuprint-python package version 0.4.25 (https://connectome-neuprint.github.io/neuprint-python/docs/).
Data analysis	Data was analyzed using a combination of open source software packages - the natverse R package (R version 4.3.1 (2023-06-16), RStudio Version 2023.06.1+524) and the accompanying fafbseg library; Google Sheets basic plotting functions; the NAVis python library version 1.3.1 (https://navis.readthedocs.io/en/latest/index.html) and accompanying fafbseg-py package version 1.13.0 (https://github.com/navis-org/fafbseg-py); seaborn version 0.11.2 (https://seaborn.pydata.org/index.html). Quantitative analyses were conducted using scipy version 1.9.1 (https://scipy.org/), networkx version 3.1 (https://networkx.org/) and scikit-learn version 1.1.2 (https://scikit-learn.org/stable/), Receptive fields were plotted using the open source packages descartes version 1.1.0 (https://pypi.org/project/descartes/), alphashape version 1.3.1 (https://pypi.org/project/alphashape/), and geopandas version 0.12.2 (https://geopandas.org/en/stable/).

For manuscripts utilizing custom algorithms or software that are central to the research but not yet described in published literature, software must be made available to editors and reviewers. We strongly encourage code deposition in a community repository (e.g. GitHub). See the Nature Portfolio guidelines for submitting code & software for further information.

Data

Policy information about availability of data

All manuscripts must include a data availability statement. This statement should provide the following information, where applicable:

- Accession codes, unique identifiers, or web links for publicly available datasets
- A description of any restrictions on data availability
- For clinical datasets or third party data, please ensure that the statement adheres to our policy

Data on visual inputs to the mushroom body that support the findings of this study are included within this paper and its Supplementary Information files. The data can also be accessed through the online Connectome Data Explorer (https://codex.flywire.ai/). All source data are provided in the accompanying Source Data files.

Research involving human participants, their data, or biological material

Policy information about studies with human participants or human data. See also policy information about sex, gender (identity/presentation), and sexual orientation and race, ethnicity and racism.

Reporting on sex and gender	This study does not include human participants or human data.
Reporting on race, ethnicity, or other socially relevant groupings	This study does not include human participants or human data.
Population characteristics	This study does not include human participants or human data.
Recruitment	This study does not include human participants or human data.
Ethics oversight	This study does not include human participants or human data.

Note that full information on the approval of the study protocol must also be provided in the manuscript.

Field-specific reporting

Please select the one below that is the best fit for your research. If you are not sure, read the appropriate sections before making your selection.

☒ Life sciences ☐ Behavioural & social sciences ☐ Ecological, evolutionary & environmental sciences

For a reference copy of the document with all sections, see nature.com/documents/nr-reporting-summary-flat.pdf

Life sciences study design

All studies must disclose on these points even when the disclosure is negative.

Sample size	Most observations in this study come from a single adult female fly (n=1 animal). Figure 7 is the only instance where we compare two samples - the FlyWire and Hemibrain connectomes (n=2 animals). This sample size was determined from the number of available connectome datasets with dense reconstructions of the relevant central brain (FlyWire and hemibrain datasets) and/or optic lobe regions (FlyWire dataset). Connectome datasets such as the FlyWire and hemibrain datasets take extensive time and resources to collect but are quality-controlled using both human and machine learning proofreading strategies, allowing us to obtain useful insights from a low sample size.
Data exclusions	We chose a priori to only include neurons in this study that made 5 or more synapses with Kenyon Cells (Direct Kenyon Cell Inputs), or 5 or more synapses with Local Visual Interneurons (Indirect Kenyon Cell Inputs). This precedent was established in Dorkenwald et al., 2023 (preprint) in the context of the FlyWire connectome in order to ensure a high degree of accuracy in the synaptic partnerships that are identified. We elected to use the 5 synapse threshold for this reason, and also to maintain consistency across FlyWire publications.
Replication	We compared neuron identities and patterns of connectivity across 3 brain hemispheres and two animals. We found consistency among the top mushroom body visual inputs and their random mode of connecting with Kenyon Cells.
Randomization	Samples could not be randomly allocated into groups because we were mostly working with data from one sample and also had no treatment conditions that required randomization.
Blinding	Blinding was not required as there was no group allocation.

Reporting for specific materials, systems and methods

We require information from authors about some types of materials, experimental systems and methods used in many studies. Here, indicate whether each material, system or method listed is relevant to your study. If you are not sure if a list item applies to your research, read the appropriate section before selecting a response.

Materials & experimental systems

n/a	Involved in the study
☒	☐ Antibodies
☒	☐ Eukaryotic cell lines
☒	☐ Palaeontology and archaeology
☐	☒ Animals and other organisms
☒	☐ Clinical data
☒	☐ Dual use research of concern
☒	☐ Plants

Methods

n/a	Involved in the study
☒	☐ ChIP-seq
☒	☐ Flow cytometry
☒	☐ MRI-based neuroimaging

Animals and other research organisms

Policy information about studies involving animals; ARRIVE guidelines recommended for reporting animal research, and Sex and Gender in Research

Laboratory animals	This study uses publicly available data collected from female adult Drosophila melanogaster. The FlyWire brain is from a 7-day-old w1118 x Canton-S G1 adult female fly (Zheng et al., 2018). The Hemibrain data is from a 5-day old w1118 x Canton S G1 adult female fly (Scheffer et al., 2020).
Wild animals	This study does not include wild animals.
Reporting on sex	Only data from female flies were used here because at the time of publication there was no male connectome data set publicly available.
Field-collected samples	This study does not include samples collected from the field.
Ethics oversight	No ethics oversight was required for this study because it consisted of analyzing publicly available datasets of invertebrate animals.

Note that full information on the approval of the study protocol must also be provided in the manuscript.

Plants

Seed stocks	This study does not contain data from plants.
Novel plant genotypes	This study does not contain data from plants.
Authentication	This study does not contain data from plants.
